# Effects of Low Temperature on Antioxidant and Heat Shock Protein Expression Profiles and Transcriptomic Responses in Crayfish (*Cherax destructor*)

**DOI:** 10.3390/antiox11091779

**Published:** 2022-09-09

**Authors:** Ying Yang, Wenyue Xu, Qichen Jiang, Yucong Ye, Jiangtao Tian, Yingying Huang, Xinglin Du, Yiming Li, Yunlong Zhao, Zhiquan Liu

**Affiliations:** 1 School of Life Science, East China Normal University, Shanghai 200241, China; 2 Freshwater Fisheries Research Institute of Jiangsu Province, 79 Chating East Street, Nanjing 210017, China; 3 School of Life and Environmental Sciences, Hangzhou Normal University, Hangzhou 311121, China; 4 School of Engineering, Hangzhou Normal University, Hangzhou 310018, China

**Keywords:** antioxidant, *Cherax destructor*, heat shock proteins, low-temperature stress, transcriptomic responses

## Abstract

Low temperature is a critical factor restricting the growth and survival of aquatic animals, but research on the mechanism of response to low temperature in *Cherax destructor* is limited. *C. destructor* is one of the most important freshwater crustaceans with strong adaptability in Australia, and it has been commercialized gradually in recent years. Here, growth indicators, antioxidant parameters, anti-stress gene expression, and transcriptome sequencing were used on crayfish following 8 weeks of low-temperature acclimation. The results showed that weight gain, length gain, and molting rates decreased as the temperature decreased. The activity of antioxidant enzymes decreased, while the content of antioxidant substances and the expression of anti-stress genes increased. Transcriptome sequencing identified 589 differentially expressed genes, 279 of which were upregulated and 310 downregulated. The gene functions and pathways for endocrine disorders, glucose metabolism, antioxidant defense, and immune responses were identified. In conclusion, although low-temperature acclimation inhibited the basal metabolism and immune ability of crayfish, it also increased the antioxidant substance content and anti-stress-gene expression to protect the organism from low-temperature damage. This study provided molecular insights into the study of low-temperature responses of low-temperature-tolerant crustacean species.

## 1. Introduction

Water temperature is an inevitable factor causing aquatic animal stress, affecting almost all physiological and biochemical processes of poikilothermic animals. It can inhibit individual growth or even lead to death at low temperatures [1,2]. When the water temperature changes beyond the tolerance temperature range of poikilothermic animals, oxygen free radicals increase, antioxidant-enzyme activity decreases, oxidative damage is aggravated, and immunity becomes suppressed in the organism [3,4]. To adapt to the drastic changes in the surrounding environment, individuals have evolved a series of temperature stress response mechanisms, such as unfolded-protein response and apoptosis [5], activation of desaturases [6,7], and increased lipid metabolism [8]. In addition, as a class of molecular chaperones, heat shock proteins can prevent protein denaturation and play a critical role in an organism’s resistance to environmental stress [9,10]. Heat shock proteins are associated with temperature tolerance and organism upregulation of *HSP* expression to protect cells from damage under temperature stress [10,11,12]. When an organism is subjected to environmental stress, *HSP70* can enhance cell viability by protecting cells from oxidative or nitrative stress, while *HSP90* can defend against pathogenic infection [13], thus improving the environmental adaptation of aquatic animals [14,15]. However, most of these studies are concentrated in species that cannot tolerate low temperature, such as *Litopenaeus vannamei* [3,16], *Marsupenaeus japonicus* [17], and *Cherax quadricarinatus* [18,19], whereas less research has been done on low-temperature-tolerant crustaceans.

*Cherax destructor* is one of the Australian freshwater crayfish with a large size, fast growth, high survival rate, and wide distribution [20]. Recently, *C. destructor* has been commercialized gradually as a potential economic species [21]. Different from other economically important Australian freshwater crayfish such as *Cherax tenuimanus* and *C. quadricarinatus*, *C. destructor* has strong temperature adaptability, and it can tolerate low temperatures [20]. At present, there are many studies on the tolerance of *C. destructor* to environmental factors such as salinity [22], dissolved oxygen [23], and pH [24], but there are fewer studies on low-temperature tolerance.

Investigating changes in gene expression patterns and metabolic pathways is the focus of understanding the molecular-response mechanisms of crustaceans under low-temperature stress. Currently, transcriptomic approaches based on high-throughput RNA sequencing (RNA-seq) have provided new analytical avenues for revealing potential genes and related pathways of organism-specific physiological processes under different environmental conditions [19,25]. RNA-seq is now widely used to study the defense responses of organisms under stress conditions. It provides comprehensive information for identifying novel immune genes and reveals potential regulatory and adaptive mechanisms [26,27,28]. With the help of RNA-seq, previous studies have identified some functional genes and physiological pathways of cold-tolerance mechanisms [29,30]. Therefore, using RNA-seq to analyze *C. destructor* at the molecular level under low temperatures is expected to help us better understand the cold-response mechanism of crayfish.

In this study, the weight growth rate and molting rate, the activity of antioxidant enzymes and glutathione content, as well as the gene expression of *heat shock proteins* (*HSPs*) and *cold shock protein* (*CPS*) in the hepatopancreas of *C. destructor* at different temperatures were examined. Transcriptome sequencing and bioinformatics analysis were applied to identify the essential genes and the major pathways in response to cold stress. The results provide a theoretical basis and valuable insights for further exploring the regulatory mechanisms of crustaceans under low-temperature stress.

## 2. Materials and Methods

### 2.1. Experimental Organisms

Healthy *C. destructor* were obtained from the Shanghai Academy of Agricultural Sciences (Shanghai, China). The temperature acclimation experiment on crayfish was carried out in culture tanks for 7 days. The tanks contained aerated water and were maintained at 20 ± 1 °C. The crayfish were fed daily with commercial feed during acclimatization, and excrement and food residue were removed.

### 2.2. Experimental Procedures

After acclimatization, the crayfish (*n* = 150) were randomly assigned to tanks (66 × 45 × 36 cm) for each temperature treatment at a density of 10 crayfish per tank. Three tanks were used for each treatment group (30, 25, 20, 15, and 10 °C). For the temperature treatment, 20 °C was maintained until the end of sampling. For the other four temperature treatments, the water temperature was increased/decreased from 20 °C to 1 °C per day until the set temperature was reached. Subsequently, the temperatures were maintained at the set temperature for 8 weeks before sampling.

During cultivation, the initial body weight and initial body length were recorded, and the number of molting crayfish was recorded every day. After cultivation, final body weight and final body length were measured, and the weight gain, length gain, and molting rates were calculated. Hepatopancreas samples of *C. destructor* were rapidly removed, flash frozen in liquid nitrogen, and stored at −80 °C for transcriptomic analysis and other biochemical assays. Only the hepatopancreas from the 25 °C and 10 °C groups were used for transcriptomic analysis.

The crayfish were fed commercial feed, and the food residues and animal feces were cleaned daily. Water temperatures were checked, and the water was changed every day. The amount of water exchanged was one-third of the total, and the tank was continuously aerated.

### 2.3. Antioxidant Index Detection

The hepatopancreas tissues from the different temperature groups (30, 25, 20, 15, and 10 °C) were combined with physiological saline. The hepatopancreas weight (g)/physiological saline (mL) ratio was 1:9. Homogenate was prepared with a homogenizer on ice. The contents of total glutathione (T-GSH), reduced glutathione (GSH), and oxidized glutathione (GSSG), as well as the enzyme activity of glutathione reductase (GR) and glutathione-S-transferase (GST), were detected using commercial kits purchased from Nanjing Jiancheng Co., Ltd. (Nanjing, China). The experimental operation was carried out in strict accordance with the instructions.

### 2.4. Detection of Anti-Stress-Gene Expression

Total RNA of hepatopancreas tissues was extracted by TRIzol reagent (Aidlab, Beijing, China), following the manufacturer’s instructions. The concentration and quality of RNA were detected by a NanoDrop-2000C (Thermo Scientific, Wilmington, NC, USA) and 1% agarose gel electrophoresis, respectively. Total RNA first-strand cDNA of each sample was generated using the PrimeScript™ RT Master Mix Real Time Kit (Takara, Japan). The transcribed cDNA was stored at −20 °C for subsequent experiments. The fluorescent quantification dye was TransStart Top Green qPCR SuperMix (TransGen, Beijing, China). Primer sequences for anti-stress genes are shown in Table S1. *18S rRNA* was used as an internal reference gene, and the 2^−ΔΔct^ method was used for calculation [31].

### 2.5. Transcriptomic Analysis

#### 2.5.1. Transcriptome Sequencing

Total RNA from hepatopancreas tissues of *C. destructor* in the 25 °C and 10 °C groups was extracted using the mirVanaTM miRNA Isolation Kit (Ambion, Austin, TX, USA). An Agilent 2100 Bioanalyzer (Agilent Technologies, Santa Clara, CA, USA) and a NanoDrop 2000 spectrophotometer (Thermo Scientific) were used to analyze the RNA integrity and quality, respectively. The TruSeq Stranded mRNA LT Sample Prep Kit (Illumina, San Diego, CA, USA) was used to construct cDNA libraries, following standardized kit procedures. The libraries were sequenced on the Illumine sequencing platform (Illumina HiSeq X Ten) at Shanghai OE Biotech Co., Ltd. (Shanghai, China), and 150 bp paired-end reads were generated.

#### 2.5.2. RNA-Seq Read Processing and Mapping

The raw image data obtained by high-throughput sequencing were converted into raw sequence data by base calling analysis. They contained the sequence information of the raw sequence data and the sequencing quality information. Trimmomatic software [32], which controls the quality of raw data, including removal of adapters, low-quality reads, and low-quality bases, was utilized as it also obtains high-quality clean reads. Clean reads were mapped to the reference genome of *C. destructor* using HISAT2, and every sample was independently mapped. The software parameters were default values.

#### 2.5.3. Differentially Expressed Genes (DEGs) and Functional Enrichment

Htseq-count software was used to obtain the read counts of genes in each sample. Cufflinks software was used to calculate the FPMK (fragments per kb per million reads) value of each gene [33,34]. The estimates SizeFactors function of the DESeq R package was used to normalize the data, and the nbinom Test function was used to calculate the *p*-value and fold change value. Genes with *p* < 0.05 and fold change ≥ 2 were considered DEGs. M-versus-A (MA) and volcano plots were created to visualize the overall distribution of DEGs. Gene Ontology (GO) enrichment and Kyoto Encyclopedia of Genes and Genomes (KEGG) enrichment analyses were performed on DEGs to determine the biological functions and pathways that differential genes mainly affect. The GO enrichment analysis was divided into biological processes, cellular components, and molecular functions.

### 2.6. Transcriptome Data Validation

To ensure the reliability of transcriptome data, fourteen genes were randomly selected from DEGs and measured using qRT-PCR. The gene expressions were detected using the qRT-PCR method, described in Section 2.4. Primer sequences for transcriptome data validation are shown in Table S2. *18S rRNA* was used as an internal reference gene, and the 2^−ΔΔct^ method was used for calculation [31].

### 2.7. Statistical Analysis

All data were presented as the mean ± standard deviation (SD). One-way analysis of variance and Tukey’s test were used to determine the differences among different temperature groups. Statistical analyses were conducted with SPSS 19.0 (IBM, Chicago, IL, USA), and graphs were constructed using Graph Pad Prism 5 (Graph Pad Software, La Jolla, CA, USA). Significant differences were indicated when *p* < 0.05.

## 3. Results

### 3.1. Effects of Temperature on the Growth Indicators of C. destructor

As shown in Table 1, the weight gain rate and length gain rate were significantly decreased in the 15 °C and 10 °C groups compared to the 30, 25, and 20 °C groups (*p* < 0.05). There was no significant difference among the 30, 25, and 20 °C groups, as well as between the 15 °C and 10 °C groups (*p* > 0.05).

### 3.2. Effects of Temperature on the Molting Rate of C. destructor

**Figure 1:**
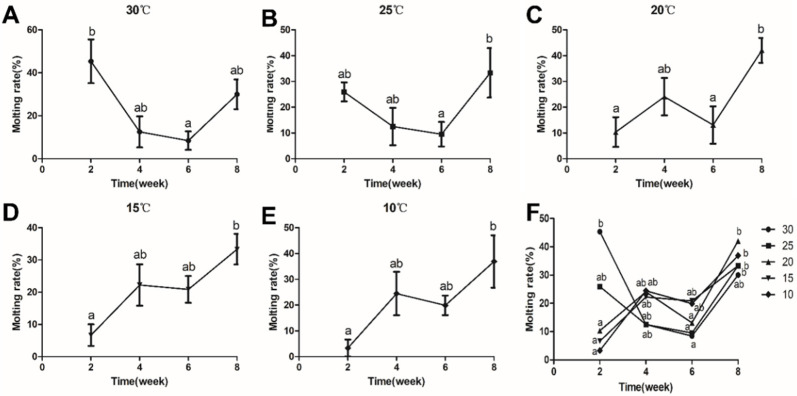
(**A**–**F**) The molting rate (%) of *C. destructor* at different temperatures (30, 25, 20, 15, and 10 °C) for 2, 4, 6, and 8 weeks. Data are presented as the mean ± SD. Means with different letters are significantly different (*p* < 0.05).

In the 30 °C and 25 °C groups, the molting rate in the second and eighth weeks was higher than in the fourth and sixth weeks. In the 20 °C group, the molting rate in the second and sixth weeks was lower than in the fourth and eighth weeks. In the 15 °C and 10 °C groups, the molting rate was enhanced as the time increased (Figure 1).

### 3.3. Effects of Temperature on Antioxidant Indicators of C. destructor

**Figure 2:**
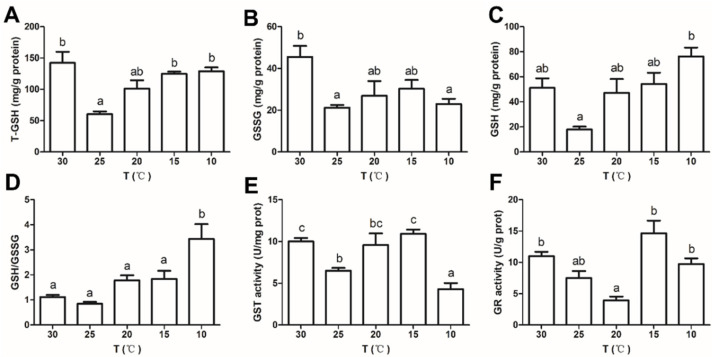
(**A**–**D**) The contents of T-GSH, GSSG, GSH, and GSH/GSSG; and (**E**,**F**) activities of GST and GR in the hepatopancreas of *C. destructor* at different temperatures (30, 25, 20, 15, and 10 °C) for 8 weeks. Data are presented as the mean ± SD of three independent experiments. Means with different letters are significantly different (*p* < 0.05).

To evaluate the antioxidant properties of *C. destructor* at different temperatures, the content of glutathione and the activities of antioxidant enzymes were detected. T-GSH had maximum values in the 30 °C group, except in the 30 °C group, T-GSH increased as the temperature decreased, and it significantly increased in the 15 °C and 10 °C groups compared with the 25 °C group (*p* < 0.05, Figure 2A). GSSG and GSH peaked in the 30 °C group and the 10 °C group, respectively, and there were no significant differences among the other temperature groups (*p* > 0.05, Figure 2B,C). GSH/GSSG had an increasing trend with the temperature decrease, and it significantly increased in the 10 °C group compared with the other groups (*p* < 0.05, Figure 2D). Except for the GST of the 10 °C group, the activity of both GR and GST first decreased and then increased as the temperature decreased. Compared with the 25 °C group, GST activity was significantly decreased in the 10 °C group (*p* < 0.05), while GR activity had no significant (Figure 2E,F).

### 3.4. Effects of Temperature on the Expression of Anti-Stress Genes in C. destructor

**Figure 3:**
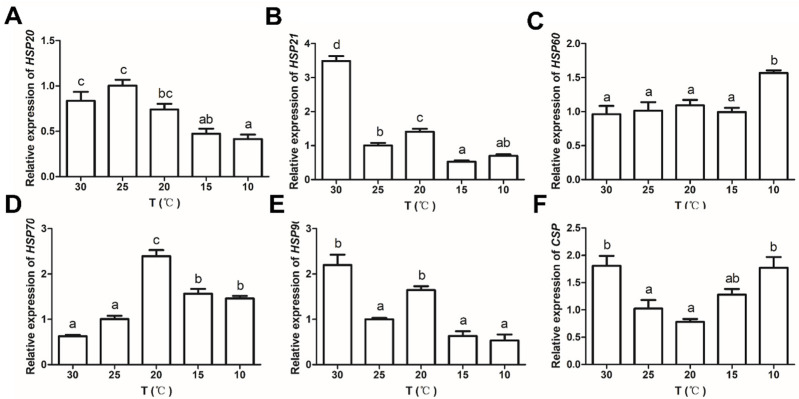
(**A**–**F**) The levels of *HSP20*, *HSP21*, *HSP60*, *HSP70*, *HSP90*, and *CSP* expression in the hepatopancreas of *C. destructor* at different temperatures (30, 25, 20, 15, and 10 °C) for 8 weeks. Data are presented as the mean ± SD of three independent experiments. Means with different letters are significantly different at *p* < 0.05.

The results of the 25 °C group are set as 1. Compared with the 25 °C group, the expression of *HSP20* significantly decreased as the temperature decreased (*p* < 0.05, Figure 3A). The expressions of *HSP21* and *HSP90* were not significantly different in the low-temperature groups compared with the 25 °C group (*p* > 0.05), while they significantly increased in the 30 °C group (*p* < 0.05, Figure 3B,E). As the temperature decreased, the expressions of *HSP60*, *HSP70*, and *CSP* significantly increased compared with the 25 °C group (*p* < 0.05), and the expression of *CSP* significantly increased in the 30 °C group (*p* < 0.05, Figure 3C,D,F).

### 3.5. GO and KEGG Pathway Enrichment Analysis

**Figure 4:**
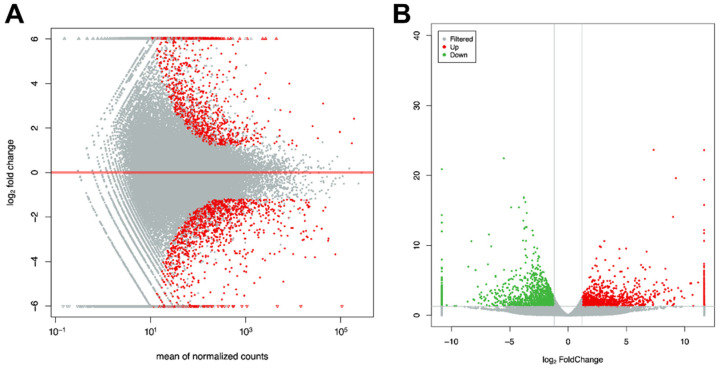
Distribution of DEGs in *C. destructor* among the low-temperature and control groups. (**A**) MA plot for DEG visualization. Each dot represents one gene, and the red dots represent DEGs. (**B**) Volcano plot map for visualization. Red and green dots represent upregulated and downregulated DEGs, respectively.

The DEGs were visualized on the MA and volcano plots (Figure 4). A total of 589 DEGs were identified, of which 279 were upregulated and 310 were downregulated.

**Figure 5:**
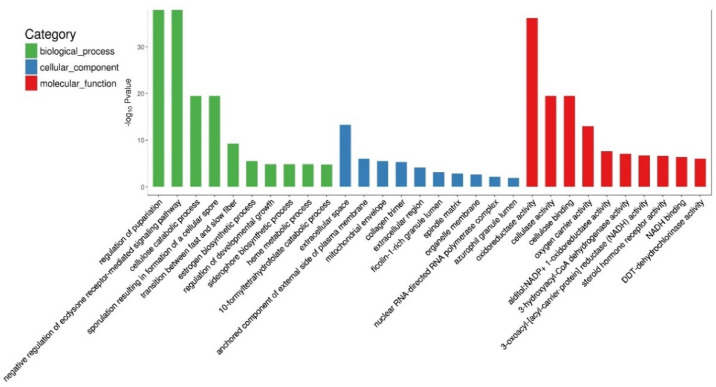
GO functional classification of DEGs in *C. destructor*. Three items are included: biological processes; cellular components; molecular functions.

To identify the key functions in crayfish affected by cold temperature, 589 DEGs were mapped into three GO items. The results showed that the top three categories of biological processes enriched by DEGs were regulation of pupariation (GO: 0106023), negative regulation of ecdysone receptor-mediated signaling pathway (GO: 0120143), and cellulose catabolic process (GO: 0030245). The top three cellular components were extracellular space (GO: 0005615), anchored component of the external side of the plasma membrane (GO: 0005615), and mitochondrial envelope (GO: 0005740). The top three molecular functions were oxidoreductase activity (GO: 0016491), cellulase activity (GO: 0008810), and cellulose binding (GO: 0030248) (Figure 5).

**Figure 6:**
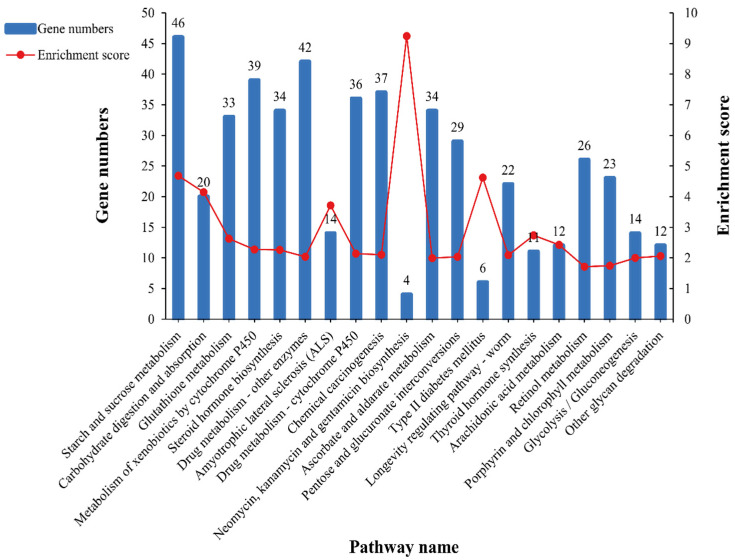
KEGG enrichment analysis of DEGs in *C. destructor*. The *x*-axis represents the pathway name. The *y*-axis represents the gene numbers enriched in the pathway (left) and enrichment score (right), corresponding to the blue bars and red lines, respectively.

As shown in Figure 6, KEGG enrichment was performed to further understand which pathways were significantly affected in the cold temperature group. The top 20 KEGG pathways that were significantly enriched were mainly involved in metabolic regulation (carbohydrate metabolism), innate immune response (glutathione metabolism, cytochrome P450 metabolism, neomycin, kanamycin, gentamicin biosynthesis, and retinol metabolism), endocrine system (steroid hormone biosynthesis and thyroid hormone synthesis), and low-temperature protective response (longevity-regulating pathway).

### 3.6. Data Validation

**Figure 7:**
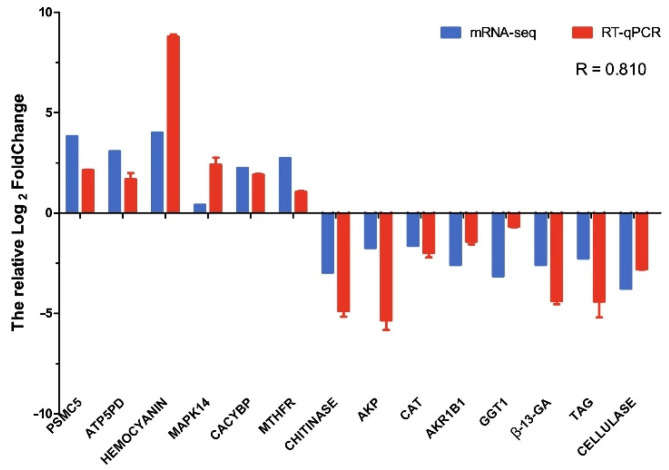
Validation of RNA-Seq results by qRT-PCR.

To verify the reliability of transcriptome sequencing results, we detected 14 randomly selected genes using qRT-PCR, as shown in Figure 7. A significant positive correlation (*R* = 0.810) between the RNA-seq results and qRT-PCR results indicates that the transcriptome results were validated.

## 4. Discussion

Changes in water temperature profoundly impact the growth and metabolism of aquatic ectothermic animals. Previous studies have shown that growth was significantly inhibited in many species at low temperatures, such as *Macrobtachium nipponense* and *C. quadricarinatus* [35,36]. Similar results were found in our experiments, where the weight growth rate and length growth rate of *C. destructor* significantly decreased in the low-temperature groups (15 °C and 10 °C) compared with other temperature groups (30, 25, and 20 °C). The results indicated that when the temperature was lower than 15 °C, the growth of *C. destructor* was inhibited. Crustacean growth is accomplished through a series of molts [37]. In the present study, as the cultivation time increased, the molting rate of *C. destructor* showed different trends in the different temperature groups. The molting rate of crayfish was lower in the 15 °C and 10 °C groups than in the other groups after two weeks of culture. The results showed that low temperature inhibited the molting rate of *C. destructor*, thus likely leading to prolonged molting intervals in crustaceans [38,39]. With the increase in cultivation time, the molting rate of crayfish in the low-temperature groups showed an upward trend, while the other groups showed a downward trend. We speculate this may be related to the prolonged molt period in low temperatures. That is, after 4–6 weeks of culture, the crayfish in the low-temperature group may gradually enter the molting period, while the other groups were in the transition between two molts. Further studies are still required to understand molting fully. These results suggested that low temperature may inhibit the growth of *C. destructor* by reducing the molting rate and prolonging the molting cycle.

To explore the physiological processes and metabolic pathways of *C. destructor* in response to low temperature, we evaluated antioxidant and anti-stress indexes at different temperatures and combined RNA-seq technology to analyze the hepatopancreas of crayfish at 25 °C and 10 °C. GO enrichment results showed DEGs were mainly enriched in biological processes related to growth and development, and molecular functions related to oxidoreductase. These results were in agreement with the growth parameters and antioxidant indicators results.

The endocrine system regulates various physiological functions of organisms to cope with environmental changes. In *Macrobrachium rosenbergii* and *L. vannamei*, changes in endocrine hormone levels were found during cold stress [40,41]. These findings confirm that the endocrine system affects individuals’ cold tolerance. In the present study, low temperature significantly altered the steroid hormone biosynthesis, including ecdysone and estrogen. Ecdysone and estrogen control molting, embryogenesis, gonadal development, and reproductive ability in aquatic animals [42,43,44]. Changes in the levels of these hormones suggest that they are involved in suppressing molting and sexual maturation at low temperatures. Responses of molting-related hormones and sexual-maturation-related hormones to temperature changes were also found in *L. vannamei* and *M. nipponense* [45,46]. Except for the steroid hormone, thyroid hormone biosynthesis was also affected in the low-temperature group. Thyroid hormone regulates the individual basal metabolic rate and is known for regulating body temperature in homothermic animals, as well as growth and development in poikilotherms [46,47,48]. It is affected by temperature in various aquatic animals [49,50,51]. Iodide is an important micronutrient in thyroid hormone biosynthesis, and iodide homeostasis within the thyroid gland is critical for thyroid hormone synthesis [52]. Iodotyrosine deiodinase is necessary to keep the balance between iodide and thyroid hormones [53]. Transcriptome results showed that the gene expression of *iodotyrosine deiodinase* was significantly down-regulated in the low-temperature group, suggesting that iodotyrosine deiodinase can be an important indicator of low temperature inhibiting thyroid hormone synthesis in crayfish. In addition, the thyroid hormone regulates thermal acclimation in the zebrafish during temperature changes [54]. Little is known about the regulatory control of thyroid hormone on thermogenesis in aquatic animals. Therefore, the thermal regulation of thyroid hormone on crayfish at low temperatures needs further exploration. The above results suggest that low temperature may lead to the synthesis and secretion of hormone disorders and then cause endocrine disorders, which finally affect individual growth and sexual maturity.

Previous studies have shown that amino acid metabolism and lipid metabolism are the main energy sources for crustaceans to cope with cold stress, and acute cold stress can lead to their dysregulation [17,55]. In the present study, among the top 20 KEGG pathways significantly enriched by DEGs under long-term low-temperature acclimation, five glucose-metabolism-related pathways (starch and sucrose metabolism, carbohydrate digestion and absorption, pentose and glucuronate interconversion, glycolysis/gluconeogenesis, and other glycan degradation) were affected. In addition, the qRT-PCR and transcriptome results showed a low-temperature-induced significant decrease in the expression of glycogenolysis-related genes, such as *β-1,3-GA*, *CELLULASE*, *amylase*, and *endoglucanase*. But the rate-limiting enzyme of glucose decomposition, *hexokinase*, was significantly upregulated. These results showed that long-term low-temperature stress could lead to abnormal glucose metabolism in individuals. Glucose is an important energy source that can be broken down by the hepatopancreas as a rapid energy source under cold stress to meet the needs of individual energy metabolism. Changes in glucose metabolism are an adaption mechanism in individuals to low temperatures; for example, *M. rosenbergii* and *L. vannamei* have increased blood glucose levels and decreased glycogen content during the adaptation process [40,55]. This result indicates the importance of a sugar source for crayfish in a long-term low-temperature environment.

The balance between the production and elimination of cellular reactive oxygen species (ROS) is disrupted when an organism is stressed. The activation of antioxidant enzymes and antioxidants is induced to prevent oxidative damage caused by excess ROS. Catalase is an antioxidant enzyme that detoxifies ROS [56]. In the transcriptome and qRT-PCR results, the expression of *CAT* in crayfish was decreased in the low-temperature group. A decrease in antioxidant enzyme activity was also observed in *Portunus trituberculatus* and *C. quadricarinatus* after low-temperature acclimation [10,18]. GSH is an antioxidant that acts on ROS [57], maintains the balance of oxidative stress and the antioxidant system, and converts GSSG to GSH via GR [58]. In *L. vannamei*, GSH has a regulatory role in temperature stress [4]. In the present study, DEGs were significantly enriched in glutathione metabolism, and the GSH content and GR activity were increased in the low-temperature group. In addition, the down-regulation of glutathione hydrolase and the up-regulation of isocitrate dehydrogenase prevent GSH breakdown and provide energy, respectively, for the GSH synthesis process [59]. They are both important enzymes that ensure GSH content. Our results showed that glutathione metabolism is involved in long-term cold acclimation. Low temperature regulates the activity of enzymes related to glutathione metabolism to increase the content of GSH, which is very important in the low-temperature adaption mechanism in *C. destructor*. Ascorbate is another antioxidant in organisms that scavenges H_2_O_2_, O_2_^−^, HO^•^ and lipid hydroperoxides [60,61]. Previous studies found that ascorbate is associated with resistance to environmental stress in aquatic animals [62,63]. The ascorbate-related pathway is significantly altered in *C. destructor* in the low-temperature group. These results suggest that long-term low temperature inhibits the activity of antioxidant enzymes but activates antioxidant-related metabolism, implying the important protective role of antioxidants during oxidative stress caused by long-term low temperature. This may be a physiological compensation mechanism of crayfish under long-term low-temperature acclimation [64].

Low temperature suppresses the immune performance of crayfish, such as antibacterial and anti-inflammatory activity. Neomycin, kanamycin, and gentamicin are from the aminoglycoside family of antibiotics [65,66,67]. They act against most gram-negative organisms and exert their antibacterial effect by blocking bacterial protein synthesis [65,68]. Retinol is involved in innate immunity and downregulates the expression of pro-inflammatory factors to exert anti-inflammatory effects [69,70]. In humans and rats, retinol has been found to have a regulatory effect at low temperatures [71,72]. In the present study, antibiotic biosynthesis and retinol metabolism were significantly changed in the low-temperature group. Mitogen-activated protein kinases (MAPKs) are involved in various physiological processes and respond to various extracellular stimuli. In *Meretrix petechialis*, *MAPK14* was activated after being challenged by *Vibrio* and elicited a series of immune responses [73]. In *Lateolabrax maculatus*, the expression of *MAPK14* was upregulated under hypoxia and salinity stress [74]. In the present study, the expression of *MAPK14* was increased in the low-temperature group, while the expression of immunity genes such as *AKP* [75], *GGT* [76], and *AKR1B1* [77] was significantly decreased. These results further confirmed that low temperature inhibited the innate immunity of crayfish. The above results indicate that the antibacterial and anti-inflammatory abilities of individuals are reduced at low temperatures and that the individual may be more susceptible to pathogen infection at this time.

Low temperature can induce a disorder of free-radical metabolism, destroy the physiological functions of cells and tissues, and cause apoptosis [19]. *HSPs* maintain homeostasis by protecting the structure and function of cells and tissues from various stressors, including temperature stress [78]. According to the molecular weight, *HSPs* can be divided into high-molecular-weight *HSPs* (i.e., *HSP100*, *HSP90*, *HSP70*, and *HSP60*) and small-molecule *HSPs* (i.e., *HSP40*, *HSP21*, and *HSP10*) [79]. In *M. rosenbergii* and *P. trituberculatus*, *HSP70* and *HSP90* were protective against temperature stress [10,13]. The expression of *HSP40* and *HSP21* was induced under temperature stress in *L. vannamei* and *C. quadricarinatus*, respectively [18,80]. In the present study, the detection of expressions for three high-molecular-weight *HSPs* (*HSP90*, *HSP70*, and *HSP60*) and two small-molecular-weight *HSPs* (*HSP21* and *HSP20*) suggest that *HSPs* with different molecular weights have different expression patterns during low-temperature adaptation. The different expression profiles were observed in the same *HSP* genes under different environmental stressors in *L. vannamei* [81], implying the necessity of different *HSP* expression patterns for individual survival. The expression of *HSP60* and *HSP70* was significantly upregulated, while other *HSPs* decreased or had no significant change at low temperatures in crayfish. The results indicated that *HSP60* and *HSP70* might have stronger protective effects at low-temperature acclimation in *C. destructor*. This may be related to the ATP dependence of high-molecular-weight *HSPs* [82].

ATP synthase is a protein that catalyzes the synthesis of ATP [83], and it provides energy for individual protective mechanisms, including *HSP*. The upregulation of ATP synthase was observed in both transcriptome and qRT-PCR results. In addition, it is well studied that *HSPs* are also involved in immune responses such as antibacterial, anti-inflammatory, and disease control [84,85]. *HSP70* is also a negative regulator of apoptosis, interfering with the occurrence of apoptosis [86,87,88]. This is related to the fact that the pathways involved in life regulation in crayfish are affected in the low-temperature group. It emphasizes the protective role of *HSPs* in crayfish at low-temperature acclimation. In transcriptome data, the longevity regulating pathway was significantly affected in the low-temperature group. Previous studies have pointed out that low temperature can improve the health and longevity of *Drosophila melanogaster* [89], *Brachionus horeanus* [90], *Caenorhabditis elegans*, and other species. The effect of temperature on the lifespan of *C. elegans* is related to *daf-16* (a key gene regulating longevity) [91]. In the present study, the expressions of *HSP60*, *SOD* (*superoxide dismutase*), *SMK-1*, and *TCERG1* (*transcription elongation regulator1*) in *C. destructor* were upregulated. SOD is an antioxidant enzyme that prevents oxidative damage. SMK-1 is required for innate immune and oxidative stress and modulates *daf-16* transcriptional specificity that regulates longevity [92]. Moreover, the overexpression of *TCERG1* extends the lifespan of *C. elegans* [93]. The increase of these genes showed low temperature could directly activate key genes that regulate lifespan in *C. destructor*. The results provide important information for the survival of cold-tolerant species at low temperatures.

*CSPs* are a class of RNA/DNA-binding proteins with one or more cold shock domains. *CSPs* are a critical factor required for cellular adaptation to low temperatures and overcoming the deleterious effects of cold stress [94,95]. Currently, increasing the expression level of *CSP* at low temperatures has been found in crustaceans such as *M. nipponense*, *C. quadricarinatus*, and *Alvinocaris longirostris* [96,97,98]. Similar results were also found in our experiments. The expression of *CSP* significantly increased in *C. destructor* in the low-temperature group, indicating *CSP* is indispensable for the survival of crayfish during cold acclimation. The specific protective mechanism of *CSP* in crustaceans is less studied and needs further exploration.

In general, stress responses are classified as primary stress responses (short term), secondary stress responses, and tertiary stress responses (long term) [99]. Primary stress responses are for the neuroendocrine system, and secondary and tertiary stress responses are for cellular responses and individual or population changes, respectively [100]. In crayfish, low-temperature acclimation affected the release of steroid hormone and thyroid hormone, and it inhibited the individual basic metabolism level and developmental process. Subsequently, a series of cellular immune responses such as antioxidant, antibacterial, anti-inflammatory, and disease resistance of crayfish were suppressed. However, during the process, anti-stress factors such as GSH, *HSP60*, *HSP70*, and *CSP* were activated to resist the deleterious effects of low temperatures. Thus, the survival of crayfish is ensured when the individual metabolism is slowed down, the molting is inhibited, and the growth is restricted.

## 5. Conclusions

This study evaluated the growth, detected the antioxidant enzyme activity and antioxidant substance content, measured the gene expression of *HSPs* and *CSP*, and used transcriptomic to explore the response mechanism at the molecular level in *C. destructor* at low temperature. The results showed that low temperature induces endocrine disorders, affects basal metabolism and glucose metabolism, inhibits antioxidant enzyme activity (GST) and immune gene expression (*AKP*, *CAT*, *GGT*, and *AKR1B1*), and slows individual growth. However, low temperatures activate the synthesis of antioxidants (GSH, antibiotics, and retinol), the expression of anti-stress genes (*HSP60*, *HSP70*, *CSP*, and *MAPK14*), and lifespan-related genes (*SMK-1* and *TCERG1*), which have important protective effects on the survival of individuals at low temperatures. Our results provide valuable information on the response metabolism of crustaceans at low temperatures.

## Figures and Tables

**Table 1:** The growth indicators of *C. destructor* at different temperatures (30, 25, 20, 15, and 10 °C) for 8 weeks.

	30	25	20	15	10
Initial crayfish wet weight (g)	3.40 ± 0.25	3.61 ± 0.32	3.47 ± 0.25	3.41 ± 0.41	3.39 ± 0.43
Initial crayfish body length (cm)	3.31 ± 0.45	3.29 ± 0.26	3.06 ± 0.40	3.32 ± 0.48	3.22 ± 0.35
Final crayfish wet weight (g)	10.86 ± 1.39	10.44 ± 0.74	9.95 ± 0.95	4.64 ± 0.26	4.46 ± 0.23
Final crayfish body length (cm)	7.95 ± 0.29	7.67 ± 0.37	7.62 ± 0.36	4.67 ± 0.53	4.63 ± 0.29
Weight gain rate (WG, %)	68.67% ^b^	65.41% ^b^	65.14% ^b^	26.40% ^a^	24.05% ^a^
Length gain rate (LG, %)	58.37% ^b^	57.08% ^b^	59.86% ^b^	28.82% ^a^	30.55% ^a^

Values are presented as the mean ± SD (*n* = 10); significant differences are indicated with different letters in the same row (*p* < 0.05).

## Data Availability

The authors declare that all data supporting the conclusions of this study are available within the article and its supplementary material.

## Supplementary Materials

The following supporting information can be downloaded at: https://www.mdpi.com/article/10.3390/antiox11091779/s1, Table S1: Primer sequences of anti-stress genes; Table S2: Primer sequences of transcriptome validation.

## References

[1] Xu D., Wu J., Sun L., Qin X., Fan X., Zheng X. (2021). Combined stress of acute cold exposure and waterless duration at low temperature induces mortality of shrimp *Litopenaeus vannamei* through injuring antioxidative and immunological response in hepatopancreas tissue. J. Therm. Biol..

[2] Ren X., Wang Q., Shao H., Xu Y., Liu P., Li J. (2021). Effects of Low Temperature on Shrimp and Crab Physiology, Behavior, and Growth: A Review. Front. Mar. Sci..

[3] Qiu J., Wang W.-N., Wang L.-j., Liu Y.-F., Wang A.-L. (2011). Oxidative stress, DNA damage and osmolality in the Pacific white shrimp, *Litopenaeus vannamei* exposed to acute low temperature stress. Comp. Biochem. Physiol. Part C Toxicol. Pharmacol..

[4] Estrada-Cárdenas P., Cruz-Moreno D.G., González-Ruiz R., Peregrino-Uriarte A.B., Leyva-Carrillo L., Camacho-Jiménez L., Quintero-Reyes I., Yepiz-Plascencia G. (2021). Combined hypoxia and high temperature affect differentially the response of antioxidant enzymes, glutathione and hydrogen peroxide in the white shrimp *Litopenaeus vannamei*. Comp. Biochem. Physiol. Part A Mol. Integr. Physiol..

[5] Wang Z., Qu Y., Yan M., Li J., Zou J., Fan L. (2019). Physiological responses of Pacific white shrimp *Litopenaeus vannamei* to temperature fluctuation in low-salinity water. Front. Physiol..

[6] Wu D.-L., Huang Y.-H., Liu Z.-Q., Yu P., Gu P.-H., Fan B., Zhao Y.-L. (2018). Molecular cloning, tissue expression and regulation of nutrition and temperature on Δ6 fatty acyl desaturase-like gene in the red claw crayfish (*Cherax quadricarinatus*). Comp. Biochem. Physiol. Part B Biochem. Mol. Biol..

[7] Wu D.-L., Rao Q.-X., Cheng L., Lv W.-W., Zhao Y.-L., Song W.-G. (2021). Cloning and characterisation of a Δ9 fatty acyl desaturase-like gene from the red claw crayfish (*Cherax quadricarinatus*) and its expression analysis under cold stress. J. Therm. Biol..

[8] Sun Z., Tan X., Liu Q., Ye H., Zou C., Xu M., Zhang Y., Ye C. (2019). Physiological, immune responses and liver lipid metabolism of orange-spotted grouper (*Epinephelus coioides*) under cold stress. Aquaculture.

[9] Chen L., Gómez R., Weiss L.C. (2021). Distinct gene expression patterns of two heat shock protein 70 members during development, diapause, and temperature stress in the freshwater crustacean *Daphnia magna*. Front. Cell Dev. Biol..

[10] Meng X.-l., Liu P., Li J., Gao B.-Q., Chen P. (2014). Physiological responses of swimming crab *Portunus trituberculatus* under cold acclimation: Antioxidant defense and heat shock proteins. Aquaculture.

[11] Kim J.H., Park H.J., Kim D.H., Oh C.W., Lee J.S., Kang J.C. (2019). Changes in hematological parameters and heat shock proteins in juvenile sablefish depending on water temperature stress. J. Aquat. Anim. Health.

[12] Zhou J., Wang L., Xin Y., Wang W.-N., He W.-Y., Wang A.-L., Liu Y. (2010). Effect of temperature on antioxidant enzyme gene expression and stress protein response in white shrimp, *Litopenaeus vannamei*. J. Therm. Biol..

[13] Ju-Ngam T., McMillan N., Yoshimizu M., Kasai H., Wongpanya R., Srisapoome P. (2021). Functional and Stress Response Analysis of Heat Shock Proteins 40 and 90 of Giant River Prawn (*Macrobrachium rosenbergii*) under Temperature and Pathogenic Bacterial Exposure Stimuli. Biomolecules.

[14] Padmini E., Rani M.U. (2008). Impact of seasonal variation on HSP70 expression quantitated in stressed fish hepatocytes. Comp. Biochem. Physiol. Part B Biochem. Mol. Biol..

[15] Pelham H.R. (1986). Speculations on the functions of the major heat shock and glucose-regulated proteins. Cell.

[16] de Souza D.M., Borges V.D., Furtado P., Romano L.A., Wasielesky W., Monserrat J.M., de Oliveira Garcia L. (2016). Antioxidant enzyme activities and immunological system analysis of *Litopenaeus vannamei* reared in biofloc technology (BFT) at different water temperatures. Aquaculture.

[17] Ren X., Yu Z., Xu Y., Zhang Y., Mu C., Liu P., Li J. (2020). Integrated transcriptomic and metabolomic responses in the hepatopancreas of kuruma shrimp (*Marsupenaeus japonicus*) under cold stress. Ecotoxicol. Environ. Saf..

[18] Wu D.-L., Liu Z.-Q., Huang Y.-H., Lv W.-W., Chen M.-H., Li Y.-M., Zhao Y.-L. (2018). Effects of cold acclimation on the survival, feeding rate, and non-specific immune responses of the freshwater red claw crayfish (*Cherax quadricarinatus*). Aquac. Int..

[19] Wu D., Huang Y., Chen Q., Jiang Q., Li Y., Zhao Y. (2019). Effects and transcriptional responses in the hepatopancreas of red claw crayfish *Cherax quadricarinatus* under cold stress. J. Therm. Biol..

[20] McCormack R.B. (2014). New records and review of the translocation of the yabby Cherax destructor into eastern drainages of New South Wales, Australia. Aust. Zool..

[21] Mauro M., Arizza V., Arculeo M., Attanzio A., Pinto P., Chirco P., Badalamenti G., Tesoriere L., Vazzana M. (2022). Haemolymphatic Parameters in Two Aquaculture Crustacean Species Cherax destructor (Clark, 1836) and Cherax quadricarinatus (Von Martens, 1868). Animals.

[22] Mills B., Geddes M. (1980). Salinty tolerance and osmoregulation of the Australian freshwater crayfish Cherax destructor Clark (Decapoda: Parastacidae). Mar. Freshw. Res..

[23] Morris S., Callaghan J. (1998). The emersion response of the Australian Yabby Cherax destructor to environmental hypoxia and the respiratory and metabolic responses to consequent air-breathing. J. Comp. Physiol. B.

[24] Ellis B., Morris S. (1995). Effects of extreme pH on the physiology of the Australian’yabby’Cherax destructor: Acute and chronic changes in haemolymph oxygen levels, oxygen consumption and metabolic levels. J. Exp. Biol..

[25] Chen K., Li E., Li T., Xu C., Wang X., Lin H., Qin J.G., Chen L. (2015). Transcriptome and molecular pathway analysis of the hepatopancreas in the Pacific white shrimp *Litopenaeus vannamei* under chronic low-salinity stress. PLoS ONE.

[26] Li Y., Zhou F., Huang J., Yang L., Jiang S., Yang Q., He J., Jiang S. (2018). Transcriptome reveals involvement of immune defense, oxidative imbalance, and apoptosis in ammonia-stress response of the black tiger shrimp (*Penaeus monodon*). Fish Shellfish Immunol..

[27] Zhong S., Mao Y., Wang J., Liu M., Zhang M., Su Y. (2017). Transcriptome analysis of Kuruma shrimp (*Marsupenaeus japonicus*) hepatopancreas in response to white spot syndrome virus (WSSV) under experimental infection. Fish Shellfish Immunol..

[28] Huang W., Li H., Cheng C., Ren C., Chen T., Jiang X., Cheng K., Luo P., Hu C. (2018). Analysis of the transcriptome data in *Litopenaeus vannamei* reveals the immune basis and predicts the hub regulation-genes in response to high-pH stress. PLoS ONE.

[29] Lou F., Gao T., Han Z. (2019). Transcriptome analyses reveal alterations in muscle metabolism, immune responses and reproductive behavior of Japanese mantis shrimp (*Oratosquilla oratoria*) at different cold temperature. Comp. Biochem. Physiol. D-Genom. Proteom..

[30] Li Y., Wang J., Jin Y., Ji G., Zhang X. (2022). Significant genes in response to low temperature in *Penaeus chinensis* screened from multiple groups of transcriptome comparison. J. Therm. Biol..

[31] Livak K.J., Schmittgen T.D. (2001). Analysis of relative gene expression data using real-time quantitative PCR and the 2−ΔΔCT method. Methods.

[32] Bolger A.M., Lohse M., Usadel B. (2014). Trimmomatic: A flexible trimmer for Illumina sequence data. Bioinformatics.

[33] Roberts A., Trapnell C., Donaghey J., Rinn J.L., Pachter L. (2011). Improving RNA-Seq expression estimates by correcting for fragment bias. Genome Biol..

[34] Trapnell C., Williams B.A., Pertea G., Mortazavi A., Kwan G., Van Baren M.J., Salzberg S.L., Wold B.J., Pachter L. (2010). Transcript assembly and quantification by RNA-Seq reveals unannotated transcripts and isoform switching during cell differentiation. Nat. Biotechnol..

[35] Wang W.-N., Wang A.-L., Liu Y., Xiu J., Liu Z.-B., Sun R.-Y. (2006). Effects of temperature on growth, adenosine phosphates, ATPase and cellular defense response of juvenile shrimp *Macrobrachium nipponense*. Aquaculture.

[36] García-Guerrero M., Hernández-Sandoval P., Orduña-Rojas J., Cortés-Jacinto E. (2013). Effect of temperature on weight increase, survival, and thermal preference of juvenile redclaw crayfish *Cherax quadricarinatus*. Hidrobiológica.

[37] Nagasawa H. (2012). The crustacean cuticle: Structure, composition and mineralization. Front. Biosci..

[38] Gong J., Yu K., Shu L., Ye H., Li S., Zeng C. (2015). Evaluating the effects of temperature, salinity, starvation and autotomy on molting success, molting interval and expression of ecdysone receptor in early juvenile mud crabs, *Scylla paramamosain*. J. Exp. Mar. Biol. Ecol..

[39] Travis D.F. (1954). The molting cycle of the spiny lobster, *Panulirus argus* Latreille. I. Molting and growth in laboratory-maintained individuals. Biol. Bull..

[40] Hsieh S., Chen S., Yang Y., Kuo C. (2006). Involvement of norepinephrine in the hyperglycemic responses of the freshwater giant prawn, *Macrobrachium rosenbergii*, under cold shock. Comp. Biochem. Physiol. Part A Mol. Integr. Physiol..

[41] Pan L.-Q., Hu F.-W., Jing F.-T., Liu H.-J. (2008). The effect of different acclimation temperatures on the prophenoloxidase system and other defence parameters in *Litopenaeus vannamei*. Fish Shellfish Immunol..

[42] Subramoniam T. (2000). *Crustacean ecdysteriods* in reproduction and embryogenesis. Comp. Biochem. Physiol. Part C Pharmacol. Toxicol. Endocrinol..

[43] Nelson E.R., Habibi H.R. (2013). Estrogen receptor function and regulation in fish and other vertebrates. Gen. Comp. Endocrinol..

[44] Ye H., Huang H., Li S., Wang G. (2008). Immunorecognition of estrogen and androgen receptors in the brain and thoracic ganglion mass of mud crab, *Scylla paramamosain*. Prog. Nat. Sci..

[45] Lago-Lestón A., Ponce E., Muñoz M.E. (2007). Cloning and expression of hyperglycemic (CHH) and molt-inhibiting (MIH) hormones mRNAs from the eyestalk of shrimps of *Litopenaeus vannamei* grown in different temperature and salinity conditions. Aquaculture.

[46] Qiao H., Xiong Y., Zhang W., Fu H., Jiang S., Sun S., Bai H., Jin S., Gong Y. (2015). Characterization, expression, and function analysis of gonad-inhibiting hormone in Oriental River prawn, *Macrobrachium nipponense* and its induced expression by temperature. Comp. Biochem. Physiol. Part A Mol. Integr. Physiol..

[47] Mullur R., Liu Y.-Y., Brent G.A. (2014). Thyroid hormone regulation of metabolism. Physiol. Rev..

[48] Little A.G., Seebacher F. (2014). The evolution of endothermy is explained by thyroid hormone-mediated responses to cold in early vertebrates. J. Exp. Biol..

[49] Moriya T. (1983). The effect of temperature on the action of thyroid hormone and prolactin in larvae of the salamander *Hynobius retardatus*. Gen. Comp. Endocrinol..

[50] Politis S.N., Servili A., Mazurais D., Zambonino-Infante J.-L., Miest J.J., Tomkiewicz J., Butts I. (2018). Temperature induced variation in gene expression of thyroid hormone receptors and deiodinases of European eel (*Anguilla anguilla*) larvae. Gen. Comp. Endocrinol..

[51] Hammond S.A., Veldhoen N., Helbing C.C. (2015). Influence of temperature on thyroid hormone signaling and endocrine disruptor action in Rana (Lithobates) catesbeiana tadpoles. Gen. Comp. Endocrinol..

[52] Bizhanova A., Kopp P. (2009). The sodium-iodide symporter NIS and pendrin in iodide homeostasis of the thyroid. Endocrinology.

[53] Moreno J.C., Klootwijk W., van Toor H., Pinto G., D’Alessandro M., Lèger A., Goudie D., Polak M., Grüters A., Visser T.J. (2008). Mutations in the iodotyrosine deiodinase gene and hypothyroidism. N. Engl. J. Med..

[54] Little A.G., Kunisue T., Kannan K., Seebacher F. (2013). Thyroid hormone actions are temperature-specific and regulate thermal acclimation in zebrafish (*Danio rerio*). BMC Biol..

[55] Zhou M., Wang A.-L., Xian J.-A. (2011). Variation of free amino acid and carbohydrate concentrations in white shrimp, *Litopenaeus vannamei*: Effects of continuous cold stress. Aquaculture.

[56] Ighodaro O., Akinloye O. (2018). First line defence antioxidants-superoxide dismutase (SOD), catalase (CAT) and glutathione peroxidase (GPX): Their fundamental role in the entire antioxidant defence grid. Alex. J. Med..

[57] Hayes J.D., McLellan L.I. (1999). Glutathione and glutathione-dependent enzymes represent a co-ordinately regulated defence against oxidative stress. Free Radic. Res..

[58] Schafer F.Q., Buettner G.R. (2001). Redox environment of the cell as viewed through the redox state of the glutathione disulfide/glutathione couple. Free Radic. Biol. Med..

[59] Gálvez S., Gadal P. (1995). On the function of the NADP-dependent isocitrate dehydrogenase isoenzymes in living organisms. Plant Sci..

[60] Frei B., England L., Ames B.N. (1989). Ascorbate is an outstanding antioxidant in human blood plasma. Proc. Natl. Acad. Sci. USA.

[61] Lesser M.P. (2006). Oxidative stress in marine environments: Biochemistry and physiological ecology. Annu. Rev. Physiol..

[62] Guerriero G., Di Finizio A., Ciarcia G. (2002). Stress-induced changes of plasma antioxidants in aquacultured sea bass, *Dicentrarchus labrax*. Comp. Biochem. Physiol. Part A Mol. Integr. Physiol..

[63] Han B., Kaur V.I., Baruah K., Nguyen V.D., Bossier P. (2019). High doses of sodium ascorbate act as a prooxidant and protect gnotobiotic brine shrimp larvae (*Artemia franciscana*) against Vibrio harveyi infection coinciding with heat shock protein 70 activation. Dev. Comp. Immunol..

[64] Bullock T.H. (1955). Compensation for temperature in the metabolism and activity of poikilotherms. Biol. Rev..

[65] Sasseville D. (2010). Neomycin. Dermatitis.

[66] Umezawa H., Ueda M., Maeda K., Yagishita K., Kondō S., Okami Y., Utahara R., Ōsato Y., Nitta K., Takeuchi T. (1957). Production and isolation of a new antibiotic, kanamycin. J. Antibiot. Ser. A.

[67] Yoshizawa S., Fourmy D., Puglisi J.D. (1998). Structural origins of gentamicin antibiotic action. EMBO J..

[68] Beard E.L. (2001). The American Society of Health System Pharmacists. JONA’S Healthc. Law Ethics Regul..

[69] Emanuele E., Bertona M., Altabas K., Altabas V., Alessandrini G. (2012). Anti-inflammatory effects of a topical preparation containing nicotinamide, retinol, and 7-dehydrocholesterol in patients with acne: A gene expression study. Clin. Cosmet. Investig. Dermatol..

[70] Ruamrak C., Lourith N., Natakankitkul S. (2009). Comparison of clinical efficacies of sodium ascorbyl phosphate, retinol and their combination in acne treatment. Int. J. Cosmet. Sci..

[71] Fenzl A., Kulterer O.C., Spirk K., Mitulović G., Marculescu R., Bilban M., Baumgartner-Parzer S., Kautzky-Willer A., Kenner L., Plutzky J. (2020). Cold-mediated regulation of systemic retinol transport controls adipose tissue browning. bioRxiv.

[72] Sundaresan P., Winters V.G., Therriault D.G. (1967). Effect of low environmental temperature on the metabolism of vitamin A (retinol) in the rat. J. Nutr..

[73] Zhang S., Yu J., Wang H., Liu B., Yue X. (2019). p38 MAPK is involved in the immune response to pathogenic Vibrio in the clam *Meretrix petechialis*. Fish Shellfish Immunol..

[74] Tian Y., Wen H., Qi X., Zhang X., Li Y. (2019). Identification of mapk gene family in *Lateolabrax maculatus* and their expression profiles in response to hypoxia and salinity challenges. Gene.

[75] Lallès J.-P. (2019). Biology, environmental and nutritional modulation of skin mucus alkaline phosphatase in fish: A review. Fish Shellfish Immunol..

[76] van Beek J.H., de Moor M.H., de Geus E.J., Lubke G.H., Vink J.M., Willemsen G., Boomsma D.I. (2013). The genetic architecture of liver enzyme levels: GGT, ALT and AST. Behav. Genet..

[77] Penning T.M. (2015). The aldo-keto reductases (AKRs): Overview. Chem.-Biol. Interact..

[78] Morimoto R.I., Santoro M.G. (1998). Stress–inducible responses and heat shock proteins: New pharmacologic targets for cytoprotection. Nat. Biotechnol..

[79] Ahn Y.-J., Im E. (2020). Heterologous expression of heat shock proteins confers stress tolerance in *Escherichia coli*, an industrial cell factory: A short review. Biocatal. Agric. Biotechnol..

[80] Chen T., Lin T., Li H., Lu T., Li J., Huang W., Sun H., Jiang X., Zhang J., Yan A. (2018). Heat shock protein 40 (HSP40) in pacific white shrimp (*Litopenaeus vannamei*): Molecular cloning, tissue distribution and ontogeny, response to temperature, acidity/alkalinity and salinity stresses, and potential role in ovarian development. Front. Physiol..

[81] Qian Z., Liu X., Wang L., Wang X., Li Y., Xiang J., Wang P. (2012). Gene expression profiles of four heat shock proteins in response to different acute stresses in shrimp, Litopenaeus vannamei. Comp. Biochem. Physiol. Part C Toxicol. Pharmacol..

[82] Mallouk Y., Vayssier-Taussat M., Bonventre J.V., Polla B.S. (1999). Heat shock protein 70 and ATP as partners in cell homeostasis. Int. J. Mol. Med..

[83] Walker J.E. (2013). The ATP synthase: The understood, the uncertain and the unknown. Biochem. Soc. Transact..

[84] Srivastava P. (2002). Roles of heat-shock proteins in innate and adaptive immunity. Nat. Rev. Immunol..

[85] Pockley A.G., Henderson B. (2018). Extracellular cell stress (heat shock) proteins—Immune responses and disease: An overview. Philos. Trans. R. Soc. B Biol. Sci..

[86] Beere H.M., Green D.R. (2001). Stress management–heat shock protein-70 and the regulation of apoptosis. Trends Cell Biol..

[87] Mosser D.D., Caron A.W., Bourget L., Denis-Larose C., Massie B. (1997). Role of the human heat shock protein hsp70 in protection against stress-induced apoptosis. Mol. Cell. Biol..

[88] Ravagnan L., Gurbuxani S., Susin S.A., Maisse C., Daugas E., Zamzami N., Mak T., Jäättelä M., Penninger J.M., Garrido C. (2001). Heat-shock protein 70 antagonizes apoptosis-inducing factor. Nat. Cell Biol..

[89] Luckinbill L. (1998). Selection for longevity confers resistance to low-temperature stress in *Drosophila melanogaster*. J. Gerontol. Ser. A Biol. Sci. Med. Sci..

[90] Lee M.-C., Yoon D.-S., Lee Y., Choi H., Shin K.-H., Park H.G., Lee J.-S. (2020). Effects of low temperature on longevity and lipid metabolism in the marine rotifer *Brachionus koreanus*. Comp. Biochem. Physiol. Part A Mol. Integr. Physiol..

[91] Zhang B., Xiao R., Ronan E.A., He Y., Hsu A.-L., Liu J., Xu X.S. (2015). Environmental temperature differentially modulates *C. elegans* longevity through a thermosensitive TRP channel. Cell Rep..

[92] Wolff S., Ma H., Burch D., Maciel G.A., Hunter T., Dillin A. (2006). SMK-1, an essential regulator of DAF-16-mediated longevity. Cell.

[93] Ghazi A., Henis-Korenblit S., Kenyon C. (2009). A transcription elongation factor that links signals from the reproductive system to lifespan extension in *Caenorhabditis elegans*. PLoS Genet..

[94] Lindquist J.A., Mertens P.R. (2018). Cold shock proteins: From cellular mechanisms to pathophysiology and disease. Cell Commun. Signal..

[95] Phadtare S., Alsina J., Inouye M. (1999). Cold-shock response and cold-shock proteins. Curr. Opin. Microbiol..

[96] Sun S., Fu H., Ge X., Zhu J., Qiao H., Jin S., Zhang W. (2017). Molecular cloning and expression analysis of cold shock protein Y-box gene from oriental river pawn (*Macrobrachium nipponense*). J. Fish. China.

[97] Meng Q., Chen J., Huang Y., Jin M., Wei G., Wang W. (2012). Molecular cloning and expression analysis of the cold shock protein Y-box coding gene of red claw crayfish, *Cherax quadricarinatus*. Freshw. Fish..

[98] Hui M., Cheng J., Sha Z. (2018). Adaptation to the deep-sea hydrothermal vents and cold seeps: Insights from the transcriptomes of *Alvinocaris longirostris* in both environments. Deep Sea Res. Part I Oceanogr. Res. Pap..

[99] Wendelaar Bonga S.E. (1997). The stress response in fish. Physiol. Rev..

[100] Manfrin C., Pallavicini A., Battistella S., Lorenzon S., Giulianini P.G. (2016). Crustacean immunity: The modulation of stress responses. Lessons in Immunity.

