# Supplementary material for: Effects of Low Temperature on Antioxidant and Heat Shock Protein Expression Profiles and Transcriptomic Responses in Crayfish (Cherax destructor)
Source: Antioxidants (Basel). 2022 Sep 9;11(9):1779. doi: 10.3390/antiox11091779 (PMC9495765; doi:10.3390/antiox11091779)
Supplement: Supplementary file 1 [file antioxidants-11-01779-s001.zip › antioxidants-1882022-supplementary.pdf]

Supporting Information

Article

Effects of Low Temperature on Antioxidant and Heat Shock Protein Expression Profiles and Transcriptomic Responses in Crayfish (*Cherax destructor*)

Ying Yang [1], Wenyue Xu [1], Qichen Jiang [2], Yucong Ye [1], Jiangtao Tian [1], Yingying Huang [1], Xinglin Du [1], Yiming Li [1], Yunlong Zhao [1,*], and Zhiquan Liu [3,4,*]

[1] School of Life Science, East China Normal University, Shanghai 200241, China
[2] Freshwater Fisheries Research Institute of Jiangsu Province, 79 Chating East Street, Nanjing 210017, China
[3] School of Life and Environmental Sciences, Hangzhou Normal University, Hangzhou 311121, China
[4] School of Engineering, Hangzhou Normal University, Hangzhou 310018, China
* Correspondence: ylzhao426@163.com (Y.Z.); liuzhiquan1024@163.com (Z.L.); Tel.: +86-21-54345387 (Y.Z.), Fax: +86-21-54341006 (Y.Z.)

Table S1. Primer sequences of anti-stress genes.

Gene name	Forward sequence	Reverse sequence
HSP20	CAATAAACATCAAGAGGGAG	CAGCATAATATGGAGGAAAA
HSP21	GATCTGTTTACCGCGAGTA	CTGTTCACCCAACCAAGT
HSP60	GAGGTGGAAGTGAATGAG	AAGGCAAGGTGTCTGTAT
HSP70	CAATTTAGTTTGGTGGGTAT	CTCTTTGCCAGTTCCTTT
HSP90	ACTTCTACGATGGGCTAC	TCTGTCTGAGGGTTTCTAT
CSP	AGTTTGGCATCACTGGTA	CTTCTTCCCTTCTTCTGTT
18S	CACTCCTGCCCTACTGCT	TGGCTCCATCAAACATACC

Table S2. Primer sequences of transcriptome validation.

Gene ID	Gene name	Gene description	Forward sequence	Reverse sequence
MSTRG.14806.2	*PSMC5*	26S proteasome regulatory subunit 8	ATTATGGCAACAAATCGC	CTGGCATCATCTCAGCAA
MSTRG.13812.2	*ATP5PD*	ATP synthase subunit d	TCCCAGATCACGCAATCA	TCCTCGGCAACTTCCTTT
MSTRG.1043.1	*HEMOCYANIN*	hemocyanin	ACAGGCTGACCCTAAACA	TTCGCTGACCTCAACATC
MSTRG.7155.2	*MAPK14*	mitogen-activated protein kinase p38	GCCAAGCAACATAGCAGT	GTCAGCACCAGGAAACAG
MSTRG.7191.1	*CACYBP*	calcyclin-binding protein-like	TACCGTGACTGGAACAAT	CCAACTCTACATGACCCT
MSTRG.13395.5	*MTHFR*	One carbon pool by folate|Antifolate resistance	CCTGAAGCCACGTCTTAT	TTTATGCCAACTGCTCTAC
MSTRG.17350.2	*CHITINASE*	chitinase	GCTACTGCCACGACCAAA	TGCCTCCAACATGATACTGA
MSTRG.27970.1	*AKP*	alkaline phosphatase	AAGACTTACAGCGGAGACAA	GACAATGCCAGTAGAACGAC
MSTRG.8132.1	*CAT*	catalase	GGTGGGCAGTACCAGAGT	CAGGTGAGGTGTTGAGGC
MSTRG.22891.6	*AKR1B1*	aldo-keto reductase family 1 member B1-like	TTACAGCCTACAGTCCTCTT	TGGAATCACAATCAACCC
MSTRG.1755.1	*GGT1*	glutathione hydrolase 1 proenzyme-like isoform X1	GGCACTGTTTATGAGGAG	TGAATACTGACATTTACCG
MSTRG.9419.1	*β-1,3-GA*	beta-1,3-glucanase	GCAACCAGTATGGAGGCA	CCACCGAAGTCCCAGAAA
MSTRG.12482.1	*TAG*	triacylglycerol lipase	TGACTACCGAGGATGGCTAC	TGGAGAAAGATGGGAGGG
MSTRG.295.15	*CELLULASE*	cellulase GHF9, partial	CGCCTCCCTCGTCTACAT	CACCATACCCACTCGTTG
MSTRG.3022.1	*18S*	18S rRNA	CACTCCTGCCCTACTGCT	TGGCTCCATCAAACATACC
